# Mental health in the context of economic development

**DOI:** 10.1192/bji.2018.21

**Published:** 2018-11

**Authors:** David Skuse

**Affiliations:** Professor of Behavioural and Brain Sciences, UCL Great Ormond Street Institute of Child Health, London, United Kingdom. Email: d.skuse@ucl.ac.uk

The idea that all citizens of the world should have the opportunity to enjoy good mental health does not on the face of it seem to be controversial. Yet, as Pamela Scorza and her colleagues discuss in the first paper of our theme on global mental health issues, it took decades to persuade the United Nations to include that objective as part of its formal report on Sustainable Development Goals (SDGs) 2015–2030. Each SDG must include a target, so that progress towards the goal can be measured over the next decade or so. Within the overall Goal 3, which aims ‘to ensure healthy lives and promote well-being for all ages’, Target 3.4 refers to the promotion of mental health and well-being. The key indicator of progress is to be the proportion of people in the population with severe mental disorder who are using services. There is as yet no agreement about the appropriate target range for that indicator, and there is no mention of progress towards the mental health component of this goal in the most recent UN report on SDGs (United Nations, 2018).

Scorza and her co-authors emphasise that ensuring that the population enjoys good mental health and well-being is essential for other SDGs to be met too, such as the aim of ‘ending poverty in all its forms everywhere’ and ‘promoting sustained, inclusive and sustainable economic growth’. In a complementary article, Chee H. Ng and colleagues point out that one-third of productivity loss worldwide is attributable to mental health disorders. Arising from these observations, the Asia-Pacific Economic Cooperation forum (APEC), which comprises 21 Pacific Rim countries, has recently decided to jointly develop better and sustainable mental health systems in order to foster social inclusion and reduce other secondary consequences of mental health disorders.

To that end, a Digital Hub, which is to be the APEC coordinating centre, is being established at the University of British Columbia in Canada. The aim is to foster projects that introduce new ways of preventing mental health disorders, as well as managing them more effectively. This is an exciting development that brings with it opportunities to reflect best practice upwards, in order to influence policy makers. The forum intends to attract the attention of governments by emphasising the value to their economies of reducing psychiatric morbidity and increasing well-being in the working population. Ng expands on this theme in a further brief report, reviewing the extent to which community-based treatments and support are provided to people living in 15 of the 21 APEC countries. Once again, the emphasis is on the extent to which untreated mental health disorders are potentially detrimental to the economies of both advanced and less developed countries within the Pacific Rim region. Not surprisingly, the proportion of people suffering who do not receive effective treatment is much greater in low- and middle-income countries. APEC wishes to promote a modern recovery model of care, enabling patients to play a fulfilling role in society, while minimising if not eliminating associated stigma. Ng emphasises the critical importance of APEC in providing guidance on how funds that have been allocated to support mental health services are used most efficiently. Yet, with the best will in the world, money alone is not going to change entrenched negative sociocultural attitudes to mental illness. Battling stigma is not just a problem in Asia, as so many of our readers will know only too well.
